# The Effect of the Visual Context in the Recognition of Symbolic Gestures

**DOI:** 10.1371/journal.pone.0029644

**Published:** 2012-02-21

**Authors:** Mirta F. Villarreal, Esteban A. Fridman, Ramón C. Leiguarda

**Affiliations:** 1 Department of Cognitive Neurology, Institute for Neurological Research-FLENI, Buenos Aires, Argentina; 2 Consejo Nacional de Investigaciones Científicas y Técnicas- CONICET, Buenos Aires, Argentina; Macquarie University, Australia

## Abstract

**Background:**

To investigate, by means of fMRI, the influence of the visual environment in the process of symbolic gesture recognition. Emblems are semiotic gestures that use movements or hand postures to symbolically encode and communicate meaning, independently of language. They often require contextual information to be correctly understood. Until now, observation of symbolic gestures was studied against a blank background where the meaning and intentionality of the gesture was not fulfilled.

**Methodology/Principal Findings:**

Normal subjects were scanned while observing short videos of an individual performing symbolic gesture with or without the corresponding visual context and the context scenes without gestures. The comparison between gestures regardless of the context demonstrated increased activity in the inferior frontal gyrus, the superior parietal cortex and the temporoparietal junction in the right hemisphere and the precuneus and posterior cingulate bilaterally, while the comparison between context and gestures alone did not recruit any of these regions.

**Conclusions/Significance:**

These areas seem to be crucial for the inference of intentions in symbolic gestures observed in their natural context and represent an interrelated network formed by components of the putative human neuron mirror system as well as the mentalizing system.

## Introduction

McNeill [1] ordered human gestures according to what he has termed “Kendon's continuum”: gesticulation - to pantomimes - to emblems - to sign language. Emblems are semiotic gestures that use movement or hand postures to symbolically encode and communicate meaning. While we immediately recognize what someone else is doing when observing an emblem even in a neutral environment, the performer's intention, that is, why he/she is doing such a gesture, often requires contextual, essentially visual, information.

Two hypothetical systems are proposed to explain how people make judgments about other people's behavior, such as their goals, intentions, desires and beliefs. The putative frontoparietal human mirror neuron system (MNS) allows us to recognize the goal of perceived actions by matching it to a representation in our memory of our own actions. It rapidly and intuitively senses the other person's goal on the basis of low-level behavioral input [2]. In turn, the mentalizing system is a relatively higher cognitive level process, which provides us with the capacity to understand the other's intentions and thoughts as if we could read the other's mind [3]. It recruits cerebral regions outside the MNS, such as the superior temporal cortex, the temporoparietal junction, the posterior cingulate cortex and the medial prefrontal cortex [3]–[6].

In recent years, different researchers analyzed the intentional connotation of certain actions by manipulating their context or the way they were performed. Iacoboni et al. [7] studied the neural activity while subjects observed a hand grasping a cup within and without a visual context and presented two different ways of grasping, representing each one with a different goal. They found activity within the MNS predominantly in the right inferior frontal gyrus (IFG) when the action was performed within the context, as well as a different level of activation depending on the intention of the action. Based on these findings they suggested that MNS uses both contextual and gestural information to predict intentionality. Using repetition suppression, Hamilton and Grafton [8] found the anterior intraparietal sulcus (IPS) to be sensitive to object-directed grasping actions but not to action trajectories, suggesting that the MNS coded the immediate goals of actions rather than the kinematic properties. However, many recent studies found that the MNS explains “how” others act and “what” they do but not “why” they are doing it, which in turn could be mainly processed by the “mentalizing” network [4], [9]–[13]. For example, de Lange et al. [9] recorded neural activity while a participant observed an actor performing an ordinary or extraordinary goal-directed action in terms of its intention or its motoric manner. They found that the IFG bilaterally processes the intentionality of an observed action on the basis of the visual properties of the action, irrespective of whether the subject paid attention to the intention or not. However, when participants selectively attended the intentionality of the action, regions involved in the mentalizing system, as the right posterior superior temporal sulcus (pSTS), showed enhanced response. Mentalizing regions are also shown to be active during observation of movements that were out of context, unplanned or biomechanically impossible [4], [14]. Therefore, it seems quite reasonable to consider the interpretation of an action as a multi-componential hierarchical process, where the underlying neural substrates are shaped by learning and experience, and hierarchy is driven mainly by the required task [15].

Most brain imaging studies investigating goal inference processes based on visual cues use object-directed actions as stimuli. Emblems are studied in a variety of ways but not by contrasting them with or without the appropriate visual context. In this way, Gallagher and Frith [16] investigated the neural pathways for the perception and recognition of gestures when used to express a feeling versus a command, they discovered that the latter elicited activity in a left lateralized system associated with language and motor imitation. Lotze et al. [17] compared brain activation during observation of isolated right hand movements, body-referred movements and expressive gestures, finding that expressive gestures involved bilateral STS, medial prefrontal cortex and Broca's and Wernicke's area. Lately, we investigated the neural pathways of the recognition of symbolic or intransitive gestures in comparison with the recognition of object-oriented or transitive gestures [18] and found that symbolic gesture recognition elicited a stronger activity in the left inferior frontal area.

Up to now, there is no data about how the context influences the processing of emblematic gestures and how relevant the information the context provides is. Therefore, we studied the influence of visual context in the brain processing of symbolic gesture recognition by means of functional magnetic resonance imaging. For this purpose, we designed a paradigm where videos of the same gestures performed by an actor were presented against a blank background (GESTURE) or within their appropriate visual context (G-CONTEXT). If the contextual information provided the gesture with its full meaning and intentionality, we predicted to find activation of the MNS as well as some brain structures belonging to the mentalizing system. As the intentionality deduced in G-CONTEXT was not the only difference between the two conditions, a large visual scenario was also present; we additionally presented videos of the same scenario and actor but without performing any gesture (CONTEXT) in order to avoid visual effects in our findings.

## Materials and Methods

### 1.1 Subjects

Nineteen healthy volunteers (10 male, 9 female) aged 29.5±5.6 years were recruited for this experiment. All participants were right handed [19] and gave written informed consent approved by the Institutional Review Board of the Institute for Neurological Research-FLENI as well as in accordance with the Declaration of Helsinki.

### 1.2 Stimuli and task

**Figure 1:**
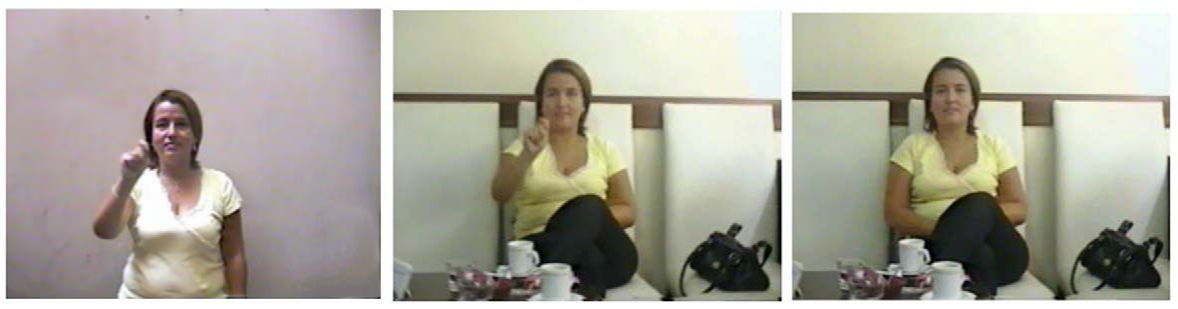
Experimental conditions. Image frames captured from some of the videos presented. Examples of one gesture (asking for the bill in a bar) in the three types of presentation: isolated, within the context and the context without the gesture (respectively from left to right).

The experiment consisted in the presentation of different videos of approximately 3 s long, depicting three different scenarios. One, called GESTURE, showed an individual performing a manual gesture against a blank background. All gestures used involved symbolic connotations like waving goodbye, hitchhiking, begging, calling a taxi, asking for a coffee in a bar or for the bill in a restaurant (the latter, being very common in our country). The second type of videos consisted of the same gestures but in this case performed in a more realistic scenario according to the meaning of the gesture, i.e. hitchhiking on the road, begging on the street. We named this category G-CONTEXT. The last group called CONTEXT showed the complex scenario with the subject in it, but in this case the person remained inactive. Three different actors performed the eighteen videos (six within each category) and each set of three videos (same gesture in the three conditions) was played by the same actor. All gestures involved were performed with the right hand and the videos were presented to the volunteer from the 3^rd^ person perspective. Each video was repeated 5 times in a pseudorandom order. In Figure 1 we show single frames of one example displaying the three conditions (permission for use of images obtained by written consent available in supporting information).

Participants were instructed to identify the action performed by the actor and to choose the correct answer according to a text screen presented one second after the clip ends. Three options were displayed: two were names of gestures (chosen among the six used) one corresponding to the right answer and the third to the word ‘nothing’. The order of the three options was counterbalanced to avoid anticipation. A fixation cross at the center of the screen was used as baseline.

Three scan sessions of 163 volumes each were acquired per subject. Each run contained 30 trials consisting in the presentation of one of the videos, followed by the text screen (3 sec) and the fixation cross within a variable time interval across trials (4–12 s, mean = 6 s). A total of 90 stimuli were presented, 30 per condition randomly distributed along the sessions. Sessions were counterbalanced across subjects.

Behaviour and latency were recorded online via a response box containing three keys for the index, middle and ring fingers. These recordings were later coded for errors and time responses. Trials with errors were discarded from the analysis.

### 1.3 Functional Magnetic Resonance Image

MRI data were acquired on a 1.5T GE HDx scanner with an 8 channel head coil. Change in blood-oxygenation-level-dependent T2* signal was measured using a gradient echo-planar imaging (EPI) sequence. Twenty four contiguous slices were taken in the AC-PC plane (TR: 2.4 s, TE: 50 ms, flip angle: 90°, FOV: 24 cm, 64×64 pixels per inch matrix, voxel size = 3.75×3.75×5). A structural MRI was acquired with the fast SPGR-IR sequence (120 slices, 1.6-mm thick slices, TR 12.956 ms, TE 6.1 ms, flip angle 15°, FOV 24 cm, 512×512 matrix). Three scans of 163 volumes were taken per subject.

### 1.4. Functional MRI Data analysis

Image processing was carried out using SPM2 (Wellcome Department of Cognitive Neurology, London, UK) implemented in MATLAB 7 (Mathworks Inc., Sherborn, MA, USA). Slice-timing correction was applied to each volume. The imaging time series was realigned to the first volume and spatially normalized to the stereotactic space of Talairach and Tournoux [20] using Montreal Neurological Institute reference brain [21]. The normalized volumes of 2×2×2 mm^3^ were spatially smoothed by an isotropic Gaussian kernel of 8 mm at full width half-maximum [22] and high pass filtered during analysis.

Individual analysis was computed using the general linear model for an event-related design including the three main conditions: GESTURE, G-CONTEXT and CONTEXT, (the text-response stimuli was computed in the design matrix but not considered in the posterior analysis). The design matrix included correction for head movements. The effects were modeled by convolving a delta function for each event type with the canonical hemodynamic response function to create regressors of interest. Individual linear contrasts were applied to the design to investigate the differential networks between conditions, and the resulting contrast images were subjected to a random effect analysis to see effects at a group level. We performed three comparisons: 1) G-CONTEXT - GESTURE; 2) CONTEXT - GESTURE and 3) G-CONTEXT - CONTEXT. From the first contrast we obtained areas related with gesture processing in a semantically congruent context but also areas related with the context per se. The second contrast was added to the analysis to observe this environmental effect, although it did not represent sufficient control for the purely visual context effect. The third contrast was proposed to separate related effects from the gesture processing alone.

Therefore, in order to isolate specific activation for gesture processing in a semantically congruent context, neither elicited by gesture processing per se or the visual characteristics of the context itself, we combined contrasts in a conjunction analysis between (G-CONTEXT – GESTURE) AND (G-CONTEXT – CONTEXT) to obtain a statistical approach. This analysis identified brain activations exclusive to G-CONTEXT that were absent in both GESTURE and CONTEXT.

Additionally, in order to disclose the individual effect of each condition we extract the BOLD beta weights from spheres of 7 mm radius built around the main coordinates of the relevant clusters (although bilaterally) that survived the last analysis. All image results were shown with an uncorrected p value of 0.001 combined with a cluster size threshold of 30 voxels. The behavioral results were put into an ANOVA for multiple comparisons, using the Bonferroni test for post hoc pair-wise comparison, implemented in the SPSS 13.0 package ©. Results for this analysis were considered significant at a level of *p* = <0.05.

## Results

### 2.1 Behavioral results

For the three conditions the error rate in answer accuracies was 3,6% and the mean reaction time (RT) in milliseconds for each condition was as follows: GESTURES 917.45±192.38; G-CONTEXT 895.91±213.34 and CONTEXT 1172.44±276.61. An ANOVA test between error rates gave a non significant result (F = 3.3, p = 0.06). For the RT, the ANOVA showed a significant main effect of F = 24.9, p<0.001 and post hoc Bonferroni test proved significant for the comparisons between GESTURE vs. CONTEXT and G-CONTEXT vs. CONTEXT with p<0.001 in both tests and no significant effect for GESTURE vs. G-CONTEXT.

### 2.2 Subtraction analysis

**Figure 2:**
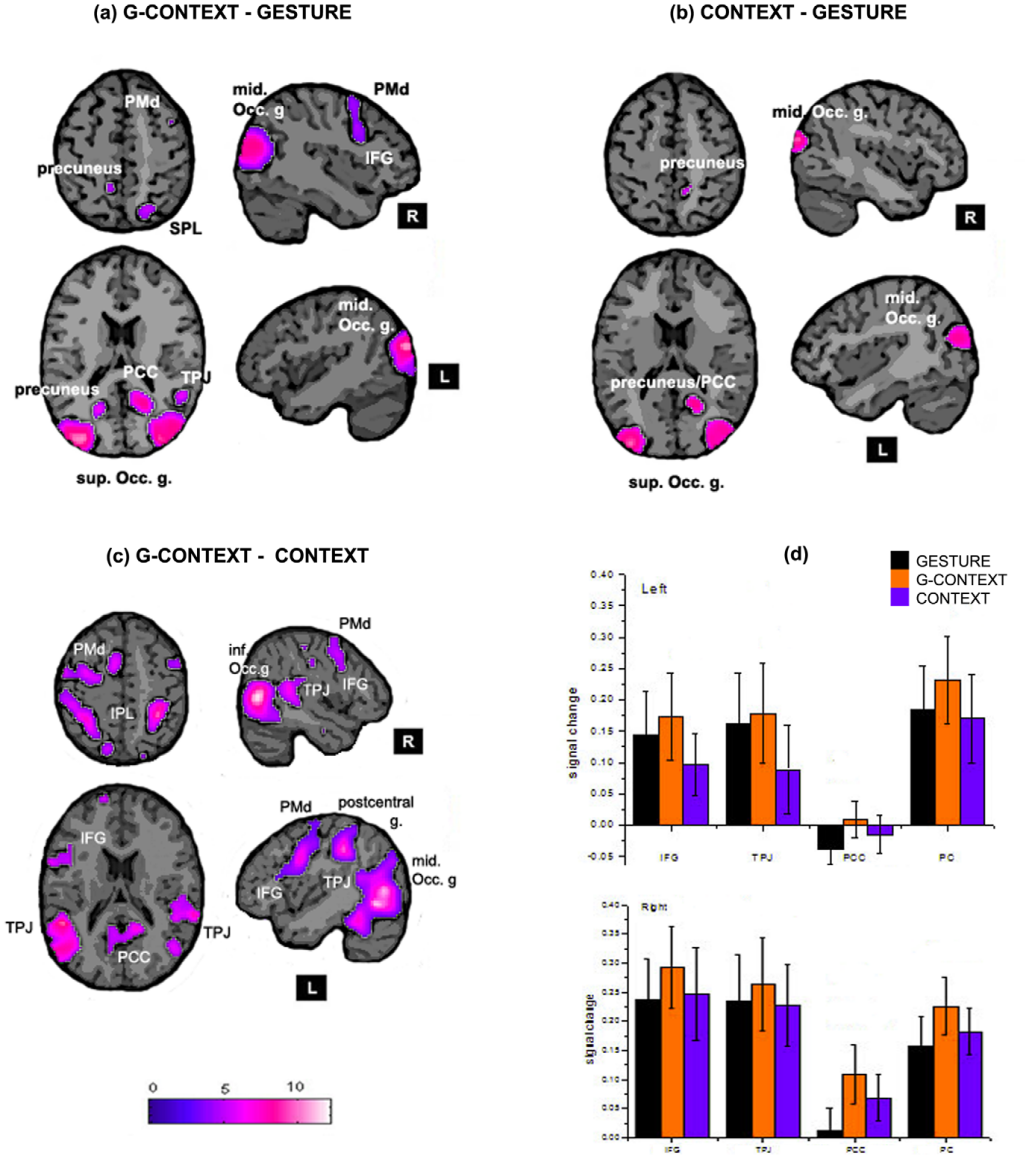
Subtraction analysis. Signal increase for (a) G-CONTEXT – GESTURE; (b) CONTEXT – GESTURE and (c) G-CONTEXT – GESTURE. The coordinates of the planes shown are z = 53 (top axial planes); z = 18 (bottom axial planes); x = −43 (top sagital planes); x = 43 (botton sagital planes). The most relevant regions are labelled. (d) BOLD signal for the three conditions and both hemispheres. In Black: GESTURE, Orange: G-CONTEXT and Blue: CONTEXT. up) Left hemisphere; down) Right hemisphere.

G-CONTEXT minus GESTURE showed active clusters within right inferior frontal gyrus (IFG), right middle frontal gyrus (MFG) and right superior parietal lobule (SPL) as well as bilateral precuneus (PC), right temporoparietal junction (TPJ), right posterior cingulate cortex (PCC) and bilateral occipital areas. CONTEXT minus GESTURE showed active clusters within bilateral occipital areas, right middle temporal gyrus, right PCC and right PC but only significative at voxel level. G-CONTEXT minus CONTEXT showed active clusters within left IFG, bilateral MFG and inferior parietal lobule (IPL), right precuneus, right PCC, bilateral TPJ and occipital areas. Figure 2a resumes the three comparisons and Table 1, the coordinates of the significant clusters.

**Table 1:** Brain regions with significant increased BOLD contrast signal in G-CONTEXT – GESTURE, CONTEXT – GESTURE and GCONTEXT-CONTEXT.

Regions (region abbreviation)	Cluster-level *P*-FWE corrected	Cluster size (voxels)	Coordinates			*t*-value
			*x*	*y*	*z*	
G-CONTEXT minus GESTURE						
Right mid. occipital gyrus	<0.001	9528	34	−86	14	6.37
Right posterior cingulate			20	−56	18	5.11
Right precuneus			22	−64	24	4.73
Right TPJ			52	−50	16	5.23
Right SPL			22	−68	54	4.51
Left sup. occipital gyrus	<0.001	5039	−40	−86	22	11.09
Left inf. occipital gyrus			−22	−92	−8	5.53
Left precuneus			−18	−62	20	4.17
Right IFG	0.023	440	38	10	30	4.26
Right PMd			42	8	54	3.84
CONTEXT minus GESTURE						
Right mid. occipital gyrus	0.032	356	20	−86	−8	7.15
Right posterior cingulate	0.028	195	20	−56	16	6.63
Right mid. temporal gyrus	0.001	732	44	−76	20	6.12
Left mid. occipital gyrus	<0.001	1161	−24	−84	−6	6.02
G-CONTEXT minus CONTEXT						
Left mid. occipital gyrus	<0.001	11292	−44	−68	4	10.13
Left TPJ			−50	−44	18	9.23
Left IFG			−48	6	30	9.09
Left IPL			−42	−32	40	8.56
Left PMd			−54	4	42	8.45
Left postcentral gyrus			−52	−26	38	6.85
Right TPJ	<0.001	3342	66	−38	18	6
Right inf. occipital gyrus			36	−92	−6	4.57
Right IPL	<0.001	4317	34	−44	56	8.61
Right postcentral gyrus			38	−34	50	8.54
Right precuneus			18	−48	40	5.42
Right posterior cingulate			−4	−58	14	4.3
Right IFG	<0.001	842	60	12	34	6.1
Right PMd			58	6	48	6

IFG: inferior frontal gyrus; PMd: dorsal premotor cortex; SPL: superior posterior parietal cortex; TPJ: temporoparietal junction.

The mean BOLD signal was greater for the G-CONTEXT condition in all regions and was higher in the right hemisphere. Figure 2.b shows the plots of the signal of each condition in all regions and both hemispheres. It is observable that GESTURE was greater than CONTEXT mainly in the left hemisphere while the opposite happened in the right hemisphere where CONTEXT was higher than GESTURE.

### 2.3 Conjunction analysis

**Figure 3:**
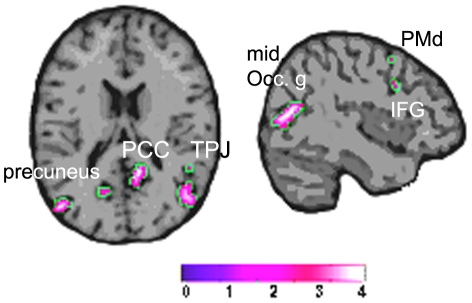
Conjunction analysis. Regions where G-CONTEXT was greater than GESTURE and CONTEXT.

The regions that survived the comparisons (G-CONTEXT – GESTURE) AND (G-CONTEXT>CONTEXT) were the right PCC, PC and TPJ and less intense the right IFG, MFG and the left PC and PCC. Activity in occipital regions was also found. Figure 3 shows the activation map and Table 2 shows the coordinates.

**Table 2:** Conjunction analysis between (GCONTEXT>GESTURE) AND (GCONTEXT>CONTEXT).

Regions (region abbreviation)	Cluster-level *P*-FWE-corrected	Cluster size (voxels)	Coordinates			*t*-value
			*x*	*y*	*z*	
Right posterior cingulate	0.037	132	14	−52	16	8.22
Left sup. occipital gyrus	0.016	183	−42	−78	26	7.26
Right TPJ	0.034	271	50	−56	16	6.93
Right precuneus	0.05	227	32	−76	34	5.4
Left posterior cingulate	* 0.001	38	−16	−62	18	5.13
Left precuneus	* 0.003		−26	−74	24	4.1
Right IFG	* 0.011	5	44	12	32	3.5

*voxel p (FDR-corr).

## Discussion

In this study, we investigated the brain network involved in the processing of symbolic gestures when immersed in their appropriate visual context. We postulated that contextual relevant information fulfils the role of completing the meaning and intentionality of the gesture (e.g.; a church or a hospital in the case of the silence gesture); and so, we predicted to find activation of brain areas within the putative human MNS in addition to some areas of the mentalizing system. To verify this hypothesis, we compared brain response when observing symbolic gestures with or without their appropriate context and found significant differences in sectors of the IFG, SPL, TPJ, and PCC, all in the right hemisphere and bilaterally in the PC with the random effect analysis. To verify whether these areas corresponded with the integration of the gesture and it's context or to environmental effects, we performed a conjunction analysis to separate the context and gesture processing effects and found that the regions that corresponded exclusively to the gesture into context condition, were the right TPJ, the PCC, the PC, the right IFG and the MFG. Furthermore, the pattern of activation for each condition obtained within the selected regions of interest revealed that G-CONTEXT presented the highest mean value in both hemispheres which was expected since all items, gesture and rich environment, were presented in this condition. In relation to the other two conditions we noticed that in some areas (right IFG, bilateral PCC and right PC) CONTEXT was higher than GESTURE, while in turn, there were other zones (left IFG, bilateral TPJ and left PC) where we found the inverse relation: GESTURE was higher than CONTEXT suggesting perhaps a gesture processing preference role within these areas. Therefore, we confirmed our prediction that when an emblematic gesture is observed within it's appropriate context, the intention behind the gesture is fully captured and so, areas belonging to the mentalizing system in addition to the putative human MNS also become active.

Right IFG was associated in previous works with intentional recognition processes besides action processing, implying a visual environment as a supplementary or complementary source of information. Related with object-directed actions, we already mentioned Iacoboni et al. [7] who compared a hand grasping action within different visual contexts and reported an enlargement of the same IFG area related with intention recognition. Another study exploiting visual scenarios for intentional purposes was done by Tylen et al. [23]. They investigated brain activity caused by a not gestural kind of communication: the way we exploit everyday objects when they are perceived as signals. As an explanation they mentioned two scenes: a cluster of flowers on the roadside and a similar bunch of flowers cut, tied and placed on the doorstep of a house. Clearly, the second scene has a social, communicative connotation: someone left the flowers for another person to find them and understand the message. Following this line of thought, they compared the brain activity of different communicative object scenarios versus other more conventional and non-communicative object scenarios. They found bilateral activity in the inferior frontal cortex predominantly on the right side only in cases with social communicative connotation. In spite of being the right IFG an area classically associated with action processing and belonging to the putative human MNS [24], [2], evidence in different kind of tasks using informative scenarios highly suggests that this region of the IFG plays a relevant role in the interpretation of the meaning of actions and their intentionality. What is not conclusive yet, is whether the visual modality of the context is activating this area or if the context itself, no matter the input channel, is the relevant task processed by this region. To answer this question further investigation is needed.

The temporoparietal junction region was associated with the representation of goals and intentions in the mentalizing system. Saxe and Powell [25] demonstrated that the right and left TPJ as well as the PCC were all recruited selectively when subjects read stories about a protagonist's thoughts or beliefs; being the right TPJ particularly specific. Furthermore, damage to this region causes selective deficits in judging the contents of other's beliefs [26]. Van Overwalle and Baetens [14] further suggested that the TPJ orients to externally generated behaviour with the aim of identifying the possible end-state of intentional behaviours. According to Ciaramidaro et al. [27] the TPJ and the right precuneus are both activated along different social intentionality dimensions, from private to social prospective intentions and conversational interactions.

We found differences within the SPL on the right side during the subtraction analysis. Previous studies have linked the SPL to the monitoring of spatial information [28] but were not reported to be associated to goals or belonging to intentional networks. Since the G-CONTEXT stimulus integrates the gesture with the appropriate environment it provides more spatial information for orienting behavior than the other conditions; therefore, more intensive activity would be expected in the SPL for this condition. We also found activation of both the PC and PCC bilaterally associated with the G-CONTEXT condition and predominantly unilaterally mainly on the right hemisphere in relation with CONTEXT condition. The PC and PCC are intimate and bilaterally interconnected providing an anatomical basis for their functional coupling [29]. The PC is also extensively connected with the lateral parietal and temporo-parietal-occipital cortices as well as with the frontal lobe [29]. The upper part of the PC is considered an area devoted to multimodal sensory integration in general [30] and visuo-spatial information processing in particular [31]. In turn, the lower part of the PC is activated in various aspects of self-representation, when ascribing social trait to others and during the interpretation of social interaction between others [32]–[34]. The PCC, together with the PC and prefrontal cortex is also active in studies exploring theory of mind. Both, the PCC and PC participate during the processing of our own intentions and consequential actions and together with the TPJ in the task of mental-state reasoning, when subject infers another person's thoughts or beliefs [11], [25], [35], [36]. As a matter of fact, Joly et al [37] demonstrated that an environment (i.e., church) raised environmental specific social forms. The PC and adjacent PCC are involved in the encoding and retrieval of spatial context of events [38] and when complex goal context need to be represented [39]. Moreover, it suggested that these areas would be part of a core network underlying a variety of cognitive functions which share the process of scenes construction, in other words, the capacity of mentally generating and maintaining a complex and coherent scene of events [40]. Therefore, it seems quite possible that the PCC and PC are both recruited by the mentalizing system to identify the situational frame and context [41] and to provide and update complex contextual associations; bilaterally, when the gesture is embedded in it's appropriate context and predominantly unilaterally (right sided) when it is inferred through a specific environment.

Consequently, it may assume that to understand the full meaning of a symbolic gesture performed in a natural context it is necessary to recruit an integral network made up by components of the putative human mirror neuron system as well as the mentalizing one; some regions of this network like the PC and PCC, seem to be predominantly recruited to provide contextual associations.
